# A Simple Index for the High-Citation Tail of Citation Distribution to Quantify Research Performance in Countries and Institutions

**DOI:** 10.1371/journal.pone.0020510

**Published:** 2011-05-27

**Authors:** Alonso Rodríguez-Navarro

**Affiliations:** Centro de Biotecnología y Genómica de Plantas, Universidad Politécnica de Madrid, Madrid, Spain; University of Maribor, Slovenia

## Abstract

**Background:**

Conventional scientometric predictors of research performance such as the number of papers, citations, and papers in the top 1% of highly cited papers cannot be validated in terms of the number of Nobel Prize achievements across countries and institutions. The purpose of this paper is to find a bibliometric indicator that correlates with the number of Nobel Prize achievements.

**Methodology/Principal Findings:**

This study assumes that the high-citation tail of citation distribution holds most of the information about high scientific performance. Here I propose the *x*-index, which is calculated from the number of national articles in the top 1% and 0.1% of highly cited papers and has a subtractive term to discount highly cited papers that are not scientific breakthroughs. The *x*-index, the number of Nobel Prize achievements, and the number of national articles in *Nature* or *Science* are highly correlated. The high correlations among these independent parameters demonstrate that they are good measures of high scientific performance because scientific excellence is their only common characteristic. However, the *x*-index has superior features as compared to the other two parameters. Nobel Prize achievements are low frequency events and their number is an imprecise indicator, which in addition is zero in most institutions; the evaluation of research making use of the number of publications in prestigious journals is not advised.

**Conclusion:**

The *x*-index is a simple and precise indicator for high research performance.

## Introduction

Scientific and technological progress is a major force in driving the economies of all developed countries. Therefore, much research has been invested to develop indicators that allow for the objective and reliable estimation of the research performance of researchers, institutions, and countries. This line of research has been especially intense since the *h*-index was proposed [1], thus giving rise to interesting studies and variants of the *h*-index [2]–[7]. Some of these new indices are the *g*-index [8], successive *h*-indices [9], *h*_w_-index [10], *h*_m_-index [11], and *e*-index [12]. Indeed, we are currently experiencing an explosion of research metrics [13], many of which have been applied to researchers and journals but probably less so to countries and institutions. However, the use of metrics to measure research performance at the country and institutional levels is more crucial, less controversial, and statistically more robust than the evaluation of researchers. Remarkably, in the absence of measurements of research performance, a country's research policy may be exclusively focused on increasing research investments, which are eventually directed toward a black box of unknown and possibly low efficiency. Low research efficiency might be particularly frequent in countries that lack a research tradition and thus are creating research systems de novo. Although the information about this possibility is limited, the research outputs of some of these countries have been investigated using different approaches [14]–[16]; in the worst-case scenario, research efficiency may be incorrectly estimated by counting the number of published papers [17].

Validation is crucial for indicators of any type [18]. However, despite the high number of correlation studies between bibliometric indicators and expert assessments that have been carried out (e.g., [19]–[22]), conventional [23] and new [24], [25] bibliometric indicators have been frequently applied to countries and institutions without explicit validations. This apparent passivity about the validation of scientific and scholarly performance metrics [18] can be explained by the difficulty of establishing numerical standards to validate indicators of research excellence or performance. This difficulty is greater in basic research in comparison with applied research, which allows the empirical treatment of questions such as whether the count of patents or patent citations may be indicative of the value of the innovation disclosed [26], [27]. In basic research it is not only difficult to measure the contribution to scientific progress but important breakthroughs occur also discontinuously, at an amazingly much lower rate than the generation of scientific data, many of which have little or no scientific impact. These low impact papers provide information that may be necessary to design the key experiments that lead to breakthroughs but if the breakthroughs are not attained the actual value of the low impact papers might be low. The seminal work of Kuhn [28] describes this discontinuous process of the scientific progress throughout history, distinguishing between normal and revolutionary science, and showing that the revolutionary ideas that modify paradigms are the engine of the scientific progress. However, when considering short periods of time, 10–20 years, the number of paradigm shifts is small and the scientific progress is driven by paradigm extensions. Although these paradigm extensions generally involve a certain number of publications, some of which are breakthrough demonstrations, the number of the key publications is very small in comparison to the total number of publications in the field.

If scientific progress is mediated by a very small part of all scientific publications the question is not only how to find an indicator for the participation of institutions and countries in this progress but also how to validate the indicator. In general terms scientific progress might be associated to high research performance or research excellence. This last concept has been extensively studied; it is considered to be complex and multidimensional, and that different indicators may reflect particular dimensions of the general concept [29]–[31]. However, in basic research the hallmark of excellence and scientific progress is the Nobel Prize. Therefore, academics perceive excellence in terms of Nobel Prize potential [30] and the number of Nobel Prize awards has been used to rank institutions ([32], [33]; ShangahaiRanking Consultancy http://www.arwu.org/). Furthermore, there is no question that Nobel Prizes are always awarded for important breakthroughs. Although these considerations lead directly to the metric of Nobel Prize achievements as an indicator of research excellence, the low frequency of these events makes their number an unsuitable indicator [34]. First, because the indicator is zero for many institutions and countries, and second, to have a sufficient number of positive cases the observation periods must be very long, which implies single measurements and imprecision. A completely different approach is to use the number of Nobel Prize achievements to validate a bibliometric indicator of much higher frequency and precision. Then the unobserved variability of a single measurement of the number of Nobel Prize achievements of a country or institution is transferred to the variability of the values of the parameter for the different cases studied. Thus, the question of whether the number of Nobel Prize achievements can be used as a standard of validation becomes a question that can be answered by statistical analysis.

This question and that of whether the most conventional bibliometric indicators can be validated in terms of the number of Nobel Prize achievements in Chemistry, Physics, and Medicine/Physiology, have been addressed previously [23]. The results of that study reveal that the number of Nobel Prize achievements can be used as a criterion of validation but that conventional bibliometric indicators such as number of papers and citations, and share of top 1% of highly cited papers cannot be validated. Interestingly, the number of national articles in *Nature* or *Science* strongly correlates with the number of Noble Prize achievements across countries and institutions. From this result, it might be incorrectly concluded that in the absence of other bibliometric indicators that correlate with the number of Nobel Prize achievements, the number of national articles in *Nature* or *Science* is the ideal indicator of scientific excellence. This conclusion is flawed because the use of the number of publications in *Nature* or *Science*, or in other prestigious journals for evaluation purposes in fact entails more problems than benefits [23]. Therefore, new bibliometric indicators for scientific excellence or high research performance that can be validated are urgently needed.

Citation distributions of scientific papers are complex and very skewed [16], [35]–[38]. Therefore, in the search for a bibliometric indicator of research performance that can be validated in terms of Nobel Prize achievements it is worth taking into account that important papers receive more citation than the average of control papers [39], [40]. In fact, potential Nobel Prize winners can be identified because nearly all Nobel laureates are highly cited within their disciplines and have produced highly cited papers [41], [42]. Consequently, it may be initially assumed that the high-citation tail of the distribution of the number of citations is the portion of the distribution that holds most of the information about scientific excellence while the rest of the distribution holds very little information. In other words, “scientific excellence ought to reveal itself in the upper tail of citation distribution functions, rather than the number of cited articles or average citation impact scores” [39]. However, the way in which this information can be transformed in a useful indicator is not evident because simple indicators of the high-citation tail such as the number of papers in the top 1% or 0.1% of highly cited papers could not be validated in terms of the number of Nobel Prize achievements [23].

In accordance with these considerations the research hypothesis of the present study is that the high-citation tail of the citation distribution holds the information about the research level of countries and institutions. As a reference of high research performance the number of Nobel Prize achievements in the time span from 1989 to 2008 can be used for validation purposes [23]. With this approach, excellence can be treated numerically and the research hypothesis can be tested by standard statistical methods. However, although this approach is simple it involves the complex question of how to transform the information of the high-citation tail in a parameter that can be treated numerically and validated in terms of Nobel Prize achievements. The present study answers this question defining an indicator of research performance from the high-citation tail of the citation distribution.

## Methods

Nobel Prize achievements are different from Nobel Prize winners in that if two or three scientists of the same country share the Nobel Prize for the same achievement, then that Nobel Prize counts as only one for the country. On the contrary, if the three scientists awarded for the same achievement are from three countries, that Nobel Prize counts as one achievement for each country [23]. The same criterion was used for institutions. The use of the number of Nobel Prize achievements instead of the number of Nobel Prize winners is consistent with the notion that the cause of a Nobel Prize laureate is an important breakthrough. Furthermore, the number of laureates adds variability to the Nobel Prize reference base (Table 1) because from one to three laureates may be awarded for the same achievement. This increase in variability is an inconvenient for the correlation analyses of this study. Nobel Prize winners were identified on Nobelprize.org (http://nobelprize.org/) and were assigned to countries or institutions as recorded in the database. All generic reference to Nobel Prize achievements refers exclusively to Nobel Prizes in Chemistry, Physics, and Physiology/Medicine.

**Table 1:** Nobel Prize achievements, *x*-index, and national articles in *Nature* or *Science* in countries and institutions.

Country or institution	Nobel Prizes	*x*-index	*Nature* or *Science*
US	57	6571	3745
Germany	7	278	292
UK	6	556	470
Japan	5	157	295
France	5	101	164
Canada	2	147	122
Switzerland	2	150	76
Australia	1	58.2	61
Sweden	1	55.9	24
Israel	1	34.5	36
Netherlands	1	153	84
Denmark	1	58.1	26
MIT	6	360	212
Stanford U	7	372	187
Zurich U	1	23.4	16
Heidelberg U	1	25.2	12
Utrecht U	1	25.9	15
Italy	0	55.8	39
Spain	0	−15.9	24

Number of Nobel Prize achievements in the period 1989–2008. The *x*-index is calculated from the mean of the yearly values of *N*_1_ and N_0.1_ from 2003 to 2007. The number of national articles in *Nature* or *Science* is the aggregate number in the same five-year period.

The Web of Science database restricted to the Science Citation Index Expanded database, and the Essential Science Indicators from Thomson Reuter's ISI Web of Knowledge (http://isiknowledge.com) were used throughout this study. To retrieve national publications for a certain country, the name of that country was introduced into the “Address” search field with the rest of the top 20 countries with the highest number of publications in the Essential Science Indicators using the Boolean Operator NOT. For institutions, the name of the institution was included with the name of the country using the Boolean Operator AND. To restrict the search to (research) articles in the “Document Type” search field, the option “Article” was selected. Similarly, to restrict the search to national articles in *Nature* or *Science*, the names of these journals were added in the “Publication Name” search field. Searches were restricted to a single year in the “Year Published” search field. The minimum number of citations needed for the publications of a certain year to belong to the percentile ranges 1%, 0.1%, and 0.01% are recorded in the percentiles table of the Baselines menu of the Essential Science Indicators. In this study, “All Fields” percentile breakdowns were used. After a search, the retrieved papers were sorted by the number of times cited, starting with the most cited paper, and the number of papers in each percentile was the order number of the last paper that met the minimum number of citations recorded in the abovementioned percentiles table shown in the Baselines menu. The total number of retrieved papers was also recorded. I report the means of the number of papers that were recorded for each year in each percentile range between 2003 and 2007 and the aggregated numbers for the national articles in *Nature* and *Science* for the same time span. The number of US national articles in a single year was over the maximum of 100,000 that the Web of Science records. Therefore, US searches were carried out in two batches, namely, papers with addresses including CA, MA, NY, IL, or MD and papers with addresses that do not include these states.

All citation data reported in this study were obtained during the month of September 2010. During the searches the Essential Science Indicators^SM^ was updated as of September 1, 2010. The dates of accesses to other URLs were September 2, 2010 for http://www.socialsciences.leiden.edu/cwts/; September 29, 2009 for http://www.scimagoir.com/; and December 10, 2008 for http://nobelprize.org/.

## Results

### Features of the high-citation tail of the citation distribution

A first observation about the high-citation tail of the citation distribution is that it contains many multinational and review papers [17], [43]–[50] in proportions that vary substantially across countries and institutions. In the countries and institutions that serve as the basis of this study (Table 1) the proportion of multinational papers in the top 1% of highly cited papers varied from 34% in US to 77% in Spain and Italy, without counting review papers. These proportions did not reflect the general proportion of multinational papers, which were 36% and 32% in Italy and Spain, respectively, versus 23% in US, excluding review papers. Thus the effect of multinational papers in the high-citation tail varied notably across countries depending on the number of national papers that were highly cited. Furthermore, the analysis of the highly cited multinational papers revealed that many of these papers involved many institutions and countries that contributed in many different ways to the result, from providing only data to assuming the scientific leadership. Therefore, an accurate assignation of the real merit of these countries in these papers was an essential prerequisite to produce a reliable indicator. For example, if a multinational paper in the top 0.1% of highly cited papers involved 50 institutions and 10 countries, it was necessary to know if the merit of a particular country in this paper was equivalent to a paper in the top 0.1% or 1% of highly cited papers or if the merit did not reach that of one in the top 1% of highly cited papers. This was obviously an impossible task. Even in papers involving two institutions from two countries, there were cases in which the connection of one of the institutions with the published study was the affiliation of a previously postdoctoral visitor to the other institution. In these cases the merit of the resulting publications was probably 90% for the hosting institution. Again, this distribution of merits cannot be easily analyzed.

In view of all these problems, I decided to continue the study excluding multinational papers, at least as a first approach that could be reconsidered depending on the results. I operated similarly with review papers because the proportion of review papers in the high-citation tail was also highly variable across countries and institutions. Furthermore, review papers amplify the citation counting of the subject of the review up to the point that the review of minor subjects might look as an important breakthrough on the basis of citation counting.

A previous observation about the tail is that some tail indicators, such as the number of papers in the top 1% or 0.1% of highly cited papers do not correlate with the number of Nobel Prize achievements when the analysis includes elite research institutions and countries publishing a large number of papers but without Nobel Prize awards [23]. To investigate this latter observation in more detail, I counted only the number of national articles in both percentiles, eliminating multinational and review papers, but the new counting did not reverse the lack of correlation. This finding suggested that the shape of the tail might be of crucial importance to quantify research performance.

**Figure 1:**
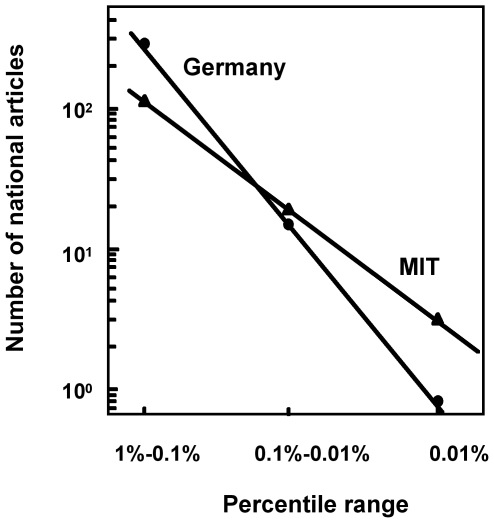
Number of national articles of Germany and MIT sorted in citation percentile ranges. The citation percentile ranges correspond to the of the world's highly cited papers. The substitution of the top 0.01% of papers for the 0.01%-0.001% range does not have practical effects. Data are means of the yearly values from 2003 to 2007.

To characterize the shape of the high-citation tail, I constructed log-log plots of the number of national articles in two citation percentile ranges, 1%-0.1% and 0.1%-0.01%, and in the top 0.01% of the highly cited papers. These plots were straight lines, which was consistent with a power law dominating the tail distribution [36]; notably, the lines showed different slopes depending on the country or institution (Figure 1 shows the plots for Germany and the MIT). Thus, apparently the same number of Nobel Prize achievements could be obtained by producing either many papers in the top 1%, and few in the top 0.01% of highly cited papers or a lower number of papers in the top 1% but higher number in the top 0.01% of highly cited papers. Consequently, the number of national articles in a single percentile did not reveal the complete account of the information about the level of research performance that the tail contains. To solve this problem, I assumed that the probability of obtaining a Nobel Prize achievement was the sum of the probabilities associated with the number of papers in several percentile ranges, which can be written as
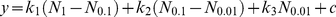
(1)where *y* is the number of Nobel Prize achievements; *N*_1_, *N*_0.1_, and *N*_0.01_ are the number of national articles in the top 1%, 0.1% and 0.01% most cited papers indexed in the ISI Web of Sciences, respectively. Intuitively, *k*_2_≥10*k*_1_ and *k*_3_≥100*k*_1_.

Although this reasoning appeared sound, the *k* constants of Equation 1 could not be fixed for Equation 1 to reasonably predict the number of Nobel Prize achievements across countries and institutions. Especially, they could not be fixed when the analysis included small countries with Nobel Prize achievements, such as Denmark and Israel and large countries without Nobel Prize winners, such as Italy and Spain. Indeed, fixing these constants was impossible because *N*_1_ and *N*_0.1_ were considerably smaller in the former than in the latter countries. This observation clearly indicated that the equation of an indicator that correlates with the number of Nobel Prize achievements must have a subtraction term dependent on both size and level of excellence of the country. In searching for the subtraction term, I investigated the type of national articles that obtains a high number of citations. These papers fell into three categories: (i) an assortment of papers that can be classified in a broad sense as method developments and statistical analyses, (ii) clinical trials, and (iii) scientific advances. A high number of citations of papers that report technical advances was noted many years ago by Eugene Garfield [51], who illustrated the issue using the Lowry method of protein measurement. The two first categories are obviously unrelated to Nobel Prize achievements and their number thus provided the obvious subtraction term to be introduced in Equation 1. However, counting these papers by inspection of the highly cited paper lists proved to be an impossible task. Therefore, the only possible estimation of the number of such papers was by modeling their production; the simplest possible model was to set this number proportional to *N*_1_ and inversely proportional to the excellence of each country's research system. Taking the *N*_1_/*N* ratio as a measure of excellence, where *N* is the total number of national articles, the subtraction term was *N*_1_ multiplied by *kN/N*_1_.

According to this model the number of Nobel Prize achievements is given by 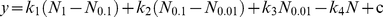
(2)


Alternatively, the excellence ratio could be used as a reduction factor in
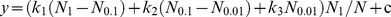
(3)


Because there are many countries and institutions with Nobel Prize achievements, the models could be tested and the *k* coefficients in Equations 2 and 3 could be obtained using multiple linear regression analysis. For this purpose, I used the data from countries with Nobel Prize achievements from 1989 to 2008, excluding US due to its outlier position, two elite research institutions, namely, MIT and Stanford University, and two countries with a high number of papers but no Nobel Prize achievements, namely, Italy and Spain [23]. Based on these data, the fit of Equation 2 was much better than that of Equation 3.

In Equation 2 the deletion of the *k*_3_ *N*_0.01_ term substantially improved the fit, probably due to the high variability of *N*_0.01_ and its low value in many countries and institutions.

### Percentile-based index of the high-citation tail

After fixing the constants in Equation 2 by multiple linear regression analysis and grouping the variables, I defined the *x*-index (excellence index) as
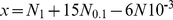
(4)


It is worth noting that the *x*-index is negative in countries and institutions in which *N*_0.1_ is zero and *N*_1_ is less than 0.6% of *N*. If *N*_0.1_ and *N*_1_ are both zero, the *x*-index is meaningless and should not be calculated.

Equation 4 was produced by a statistical approach that optimized the model described by Equation 2, but this approach did not guarantee that the model was totally correct and that the *x*-index was highly correlated with the number of Nobel Prize achievements. Therefore, the next step was to validate the *x*-index using the number of Nobel Prize achievements and national articles in *Nature* or *Science* in the countries and institutions used to fit Equation 2. Drawing on a previous study [23], I checked the inclusion of three European universities with one Nobel Prize achievement each, namely, Zurich, Heidelberg, and Utrecht (Table 1). The *x*-index was highly correlated with the number of Nobel Prize achievements. Excluding US because of their outlier position, the Pearson correlation coefficients were 0.81 (*p*<0.001) and 0.83 (*p*<0.001), excluding and including the three aforementioned European universities, respectively; the Spearmen correlation coefficients for the same groups of cases but including US were 0.88 (*p* = 0.001) and 0.85 (*p*<0.001), respectively. Considering the variability that intuitively is intrinsic to the award of a Nobel Prize these correlation coefficients seemed very high.

To assess the variability of the number of Nobel Prize achievements I divided the 20-year period of this study (Table 1) into four periods of five-consecutive years and counted the Nobel Prize achievements of Germany, UK, Japan, and France in these periods. The counts of the number of Nobel Prize achievements in the four periods were: 2-2-1-2, 0-2-2-1, 0-2-2-2, and 2-1-0-2 for the four countries, respectively. Thus in a single observation in the 1989–1993 period the distribution of the Nobel Prize achievements of these countries was: Germany and France, two; Japan and UK, zero. In contrast, in the 1999–2003 period the distribution was: Japan and UK, two; Germany, one; France, zero. To further investigate the distribution of the low frequency events that are important breakthroughs I counted the annual number of national articles in the top 0.01% of highly cited papers in several US states in five consecutive years, 2003–2007. In these counts the difference between the maximum and minimum values was approximately equal to the mean. For example, in the state of New York the numbers were: 6-7-8-2-2. These two approaches indicated that the number of Nobel Prize achievements across the cases studied in Table 1 was affected by a notable variability, which limited the maximum Pearson correlation coefficients that could be obtained between this parameter and the *x*-index. It can be reasonably estimated that even in the case of a perfect *x*-index of low variability the correlation coefficients could not be much higher than those found, 0.81 and 0.83.

As can be expected by noting the high correlation between the number of national articles in *Nature* or *Science* and the number of Nobel Prize achievements [23], the *x*-index showed a strong correlation with the number of national articles in *Nature* or *Science*. In this correlation, the Pearson correlation coefficient for all cases in Table 1 except US was 0.88 (*p*<0.001). In summary, the *x*-index, the number of Nobel Prize achievements, and the number of national articles in *Nature* or *Science* were highly correlated regardless of how the correlations were calculated.

### The *z*-index

The *x*-index estimates the total capacity of a research system to produce excellent research, which is higher in larger research systems of similar efficiency. To estimate a normalized research performance I divided the *x*-index by *N*. Thus, I defined a size-independent *z*-index as
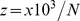
(5)


To judge the usefulness of the *x*- and *z*-indices I calculated these indices for the top 20 countries with the highest number of citations (Table 2) as well as for a sample of 20 universities of decreasing excellence (Table 3) based on both the CWTS of the Leiden University (http://www.socialsciences.leiden.edu/cwts/) and the SCIMAGO Institutions Ranking (http://www.scimagoir.com/).

**Table 2:** *x*- and *z*-indices, and number of citations per paper in a selection of countries.

Country	*x*-index	*z*-index	Citations per paper
US	6571	39.0	15.52
Switzerland	150	26.6	16.39
UK	556	16.4	15.44
Netherlands	153	15.8	15.13
Denmark	58.1	15.4	15.49
Germany	278	7.9	12.87
Canada	147	7.5	12.83
Sweden	55.9	7.1	14.40
Belgium	34.4	6.9	13.24
Israel	34.5	6.9	12.34
Australia	58.2	4.5	11.63
France	101	4.0	12.09
Japan	157	3.2	10.07
Italy	55.8	2.8	11.48
South Korea	−10.6	−0.7	6.85
China	−42.1	−0.8	5.78
Spain	−15.9	−1.0	10.18
India	−92.4	−4.8	5.54
Brazil	−55.0	−4.9	6.19
Russia	−85.5	−5.8	4.58

The 20 countries with the highest number of citations in the Essential Science Indicators of the ISI Web of Knowledge. The *x*-index is calculated from the mean of the yearly values of *N*_1_ and N_0.1_ from 2003 to 2007, and the *z*-index is the *x*-index divided by the mean of the number of national articles in these years. The number of citations per paper is taken from the Essential Science Indicators in All Fields.

**Table 3:** *x*- and *z*-indices, and indicators of the CWTS and SCIMAGO group in universities of decreasing research excellence.

University	Country	*x*-index	*z*-index	CWTS	SCIMAGO
MIT	US	360	180	2.38–2.46	2.52
Stanford U	US	372	141	2.11–1.96	2.26
U California LA	US	319	102	1.75–1.71	2.00
U Oxford	UK	114	54.2	1.67–1.63	1.89
U Cambridge	UK	106	52.9	1.70–1.63	1.88
ETH Zurich	CH	33.1	31.6	1.63–1.64	1.88
U Edinburgh	UK	35.6	28.0	1.54–1.54	1.71
U Zurich	CH	23.4	23.5	1.46–1.44	1.68
Stockholm U	SE	24.0	21.1	1.43–1.50	1.59
U Toronto	CA	63.0	20.5	1.45–1.46	1.71
U Heidelberg	DE	25.2	18.4	1.35–1.32	1.58
U Paris Sud 11	FR	18.7	17.6	1.34–130	1.40
Utrecht U	NL	25.9	17.0	1.42–1.35	1.69
U Milano	IT	32.5	16.4	1.20–1.22	1.32
K U Leuven	BE	9.8	10.4	1.35–1.38	1.54
Seoul National U	SK	24.3	9.6	1.03–1.03	1.08
U Melbourne	AU	18.6	9.6	1.34–1.26	1.50
U Barcelona	ES	6.1	2.5	1.24–1.19	1.35
Peking U	CN	3.1	1.5	1.05–0.94	0.96
U Complutense Madrid	ES	−2.6	−2.5	0.93–0.93	1.07

The *x*-index is calculated from the mean of the yearly values of *N*_1_ and N_0.1_ from 2003 to 2007, and the *z*-index is the *x*-index divided by the mean of the number of national articles in these years. *MNCS2-CPPFCSm* indicators of the CWTS of the Leiden University (http://www.socialsciences.leiden.edu/cwts/) and Field Normalized Citation Score of SCIMAGO (http://www.scimagoir.com/).

Regarding the *z*-index values in Table 2, the high performances of US in the world and of Switzerland in Europe were evident. Countries with negative values were countries that have developed their research system in the last 25 years, except Russia. Some of these countries showed a high increase in the number of national articles during the five-year window of the study, from 2003 to 2007, as well as great variability in the annual values of the *x*-index. The most notable case was China, in which *N* in 2007 was 2.1 times higher than in 2003 (71,090 and 33,815, respectively); in South Korea the increase was 52% over the same period. This rapid increase might be incompatible with keeping high research excellence. Therefore, for the assessment of the actual scientific level of these countries, the study of the oldest universities, in which the increase in production is probably slower, might be necessary.

The *z*-index decreased simultaneously with the number of citations per paper in countries (Table 2) and with the CWTS and SCIMAGO indicators in universities (Table 3), with minor discrepancies. In contrast, the total variation of the *z*-index was much higher than that for any of the other indicators.

In a previous paper [23] I did not test the validation of the *h*-index for countries and institutions in terms of Nobel Prize achievements (for other types of validations see [52]). The data summarized in Table 4 for a selection of countries and institutions demonstrate that the *h*-index cannot be validated in terms of the number of Nobel Prize achievements. Notably, according to the *h*-index it seems that Italy produces the same amount of excellent research than MIT, which is not the case according to the number of Nobel Prize achievements. The *h*-index did not correlate with either the size-dependent *x*-index or the size-independent *z*-index.

**Table 4:** Nobel Prize achievements and *h*-, *x*-, and *z*-indices in a selection of countries and institutions.

Country or institution	Nobel Prize achievements	*h*-index	*x*-index	*z*-index
Germany	7	207–128	278	7.9
Stanford U	7	153–102	372	141
MIT	6	146–101	360	180
Japan	5	201–121	157	3.2
Canada	2	176–101	147	7.5
Denmark	1	99–68	58	15.4
Italy	0	141–95	56	2.8
Spain	0	105–80	−16	−1.0

The *h*-index is calculated from national articles in years 1995 and 2005, first-second data, respectively. The *x*- and *z*-indices are taken from Tables 1, 2, and 3.

## Discussion

The *x*-index described here is a percentile-based indicator for the 1% highly cited tail of the distribution of citations to research publications, which has been specifically formulated to estimate the level of research performance of countries and institutions. Two terms of the formula, the numbers of national articles in the top 1% and 0.1% of highly cited papers are simple bibliometric parameters that are intuitively associated with research excellence. In fact, the share of the top 1% of highly cited papers has been previously used to rank countries as a function of the quality of their scientific research [53]. Because the calculation of the *x*-index involves a large number of papers its value is high with reference to the number of Nobel Prize achievements, a mean of 1,170 for a country or institution with one Nobel Prize achievement per year (calculated from data in Table 1).

The main characteristic of the formula of the *x*-index is that it was specifically designed to maximize the correlation of the *x*-index with the number of Nobel Prize achievements. The final results are correlation coefficients of 0.81–0.88, depending on the calculation procedure, with *p* values less than 0.001. These correlation coefficients are very high considering that one of the variables of the correlation, the number Nobel Prize achievements, corresponds to events of low frequency that depends on many factors. The number of achievements was counted in a period of 20 years because in shorter periods the number of the studied countries would be too low [23]. This long period implies that measurements could not be repeated and, consequently, that the unobserved variability of the single measurement of the number of Nobel Prize achievements in each country or institution is transferred to the variability of the values of the parameter across countries and institutions, which decreases the correlation coefficient. Two approaches to assess this variability indicated that Pearson correlation coefficients of 0.81–0.83 are close the highest that can be expected. Consequently, the *x*-index is unquestionably validated in terms of the number of Nobel Prize achievements.

The *x*-index formula has two singular features, the exclusion of multinational and review papers, and the subtraction term. The basis of the exclusion of review papers and for creating the subtraction term is conceptual because there are many highly cited papers that are not scientific breakthroughs. In contrast the exclusion of multinational papers is exclusively operational. Indeed, if it had been possible to assign the real merits that each country or institution has in these papers I would not have excluded them. The key issue is that I could not find an index for the high-citation tail that correlates with the number of Nobel Prize achievements if multinational papers were counted with the same weight for all countries involved. Therefore, the arising question is why an index excluding multinational papers can be validated in terms of the number of Nobel Prize achievement. Although correlation analysis does not normally provide information about causality, the present case is slightly different. The remarkable result is that the correlation coefficient that validates the *x*-index is so high, considering the variability of the number of Nobel Prize achievements, that a hypothetic index including the merits in multinational papers could not be appreciably higher. This fact has two possible mathematical explanations, either the merit of multinational papers is negligible or it is proportional to the *x*-index. Because the former possibility seems to be incompatible with the large number of highly cited multinational papers, the latter must be the correct explanation, and this is not surprising. Certainly the proportion of multinational articles in the top 1% of highly cited papers is very different across countries, e.g. 34% in US and 77% in Spain, but the merits of these countries in the highly cited papers are also very different. I have already explained that to include the merits of highly cited multinational papers in the *x*-index their number must be transformed into equivalents of top 1% and 0.1% of highly cited papers considering the merits of each participating country. I found that in countries with less competitive research the proportion of multinational papers in the top 1% of highly cited papers is very high because the number of national highly cited papers is very low. Therefore, it can be expected that in these countries the abovementioned transformation into equivalents of top 1% and 0.1% of highly cited papers would lead to a drastic reduction of the original number of highly cited papers. Indeed, it seems inconceivable that a country is highly competitive in multinational papers and poorly competitive in national papers. In the example above, the US research superiority both in terms of scientific leadership and of the number of institutions participating in multinational papers suggests that the transformation would not reduce the untransformed number of multinational articles very much. The opposite applies to Spain, in which the equivalents of top 1% and 0.1% of highly cited papers could be many times lower than the untransformed numbers.

The number of national articles in *Nature* or *Science* correlates with the number of Nobel Prize achievements [23] and with the *x*-index. However, for general evaluation purposes, the *x*-index is a better indicator than the number of national articles in *Nature* or *Science*. In the first place to be consistent with the basic ideas of this study because all journals publish papers that receive a low number of citations that should not be counted as excellent. The second reason is practical because evaluating research by the number of publications in any prestigious journal would bring more problems than benefits. This type of evaluation is problematic with respect to the journals themselves, because the pressure on the researchers to increase the value of the criterion would result in an unnecessary increase of submissions to the journals, while at the country level, researchers might only achieve publication of low-cited papers in highly cited journals [17], which should not be a scientific target.

The *x*-index calculated in this study used the “All Fields” percentile breakdowns of the Essential Science Indicators. This simplification is used here because I only try to illustrate and validate the method. For evaluation purposes, it may be used or not, depending on whether the institution under evaluation carries out research in all major research fields (e.g., universities) or it is specialized in a single field (e.g., cancer research centers). In the former case the simplification can be used, but it is obvious that fields with higher numbers of citations will have more influence on the *x*-index than the fields with lower numbers of citations. However, this problem may not be very important. For example, the 1% breakdowns in 2007 for All Fields, Biology & Biochemistry, Chemistry, and Physics were: 52, 63, 54, and 43 citations, respectively. By using only the breakdown of 52 citations, the index uses a percentile that is slightly higher than 1% for Physics and slightly lower than 1% for Biology & Biochemistry. These deviations seem irrelevant in comparison with the dramatic differences in the *x*-index across countries and institutions, but more importantly, the high correlation of the *x*-index with the number of Nobel Prize achievements demonstrates that the approach is appropriate. The issue is different in institutions doing research in a single field in which its percentile breakdowns are very different from those of the “All Fields”, for example, Molecular Biology & Genetics. In those cases, the specific percentile breakdowns should be used. Independent of these considerations, the *x*-index can be calculated for a specific research field without the interference of other research fields because the ISI Web of Science allows the inclusion of journal titles in search queries. By selecting the journals, different research fields can be selected.

A notable characteristic of the *x*-index is that because of its subtraction term, it can be negative in countries and institutions with a low proportion of *N*_1_ with respect to *N*, which normally implies that *N*_0.1_ is zero. This characteristic precludes the possibility of including these countries or institutions in proportional rankings (i.e., the index is proportional to the probability of obtaining a Nobel Prize award) together with institutions having positive *x*-indices. However, this problem is a small price to pay for the simplicity of the index. The subtraction term might be eliminated by using more complex models than that used to formulate Equation 2. However, complex indices might not be necessary. The *x*-index is an indicator of research level, and a negative value of the index clearly indicates that the level is low. To quantify the probability that a country with a negative *x*-index obtains a Nobel Prize award in comparison with leading research countries seems a minor issue.

The *x*-index cannot be calculated when *N*_1_ and *N*_0.1_ are zero, which occurs in many institutions around the world. The ranking of these institutions might be achieved by creating a new index using the number of national articles in the top 10% of highly cited papers. However, the need for this new index is not urgent, except for very small institutions. Institutions in which *N* is 500 or higher and *N*_1_ is zero over five successive years have a low level of scientific excellence. To quantify the probability that such institutions obtain a Nobel Prize award seems of little practical interest.

The *z*-index provides information about the intrinsic level of the research performance in countries and institutions, thus allowing the comparison of research systems of different sizes. The purpose of this report is to illustrate a method rather than to make country or institution comparisons, and the selected cases recorded in Tables 2 and 3 only try to show the capacity of the *x*- and *z*-indices to provide insight into differences among countries and institutions. For example, the world leadership of US [54] and the European leadership of Switzerland in research are clearly demonstrated by the *z*-index, independently of the dramatic difference in the sizes of these two countries. In countries, the *z*-index decreases simultaneously with the number of citations per paper recorded in the Essential Science Indicators of the ISI Web of Knowledge, but the number of citations per paper shows a more attenuated change (Table 2). This response can be at least partially explained by the effect that the number of citations of the papers in the high-citation tail has on the mean of the number of citations of all papers [55]. If this explanation is correct, the evaluation of scientific excellence by the mean number of citations of all publications is only an attenuated evaluation of the high-citation tail. Regarding both the number of citations per paper and the *z*-index, it is worth emphasizing that technological papers receive lower number of citations than scientific papers. Therefore, countries with high proportions of technological versus scientific research might be undervalued with a general *z*-index. The calculation of the *x*- and *z*-indices by research fields solves this problem.

In institutions, the *z*-index varies simultaneously with the CWTS and SCIMAGO indicators (Table 3), which are based on the total number of publications. In contrast with countries (Table 2), because many institutions are very similar in size, the *x*- and *z*-indices vary almost in parallel. Therefore, comparisons of these indices with the CWTS and SCIMAGO indicators illustrate better than in the case of countries the quality of the information provided by the *x*- and *z*-indices. Considering only positive values, the *z*-index varies more than 100 times where the other indicators vary 2.5 times (Table 3). For example, in universities with one versus six or seven Nobel Prize achievements in Table 1, the CWTS and SCIMAGO indicators vary less than 1∶2, while the *z*-index varies a minimum of 1∶6 (Table 3); the differences are larger when institutions with low levels of excellence are compared to leading research institutions. For example, for a hypothetical university similar in size to MIT with an annual production of 2,000 national articles, 14 in the top 1% and none in the top 0.1% highly cited papers, the *x*- and *z*-indices would be 2 and 1, respectively; the CWTS and SCIMAGO indicators may be around 1.0 (see Table 3). Focusing on MIT, the CWTS and SCIMAGO indicators lead to the obviously erroneous conclusion that the MIT would promote the advancement of science only 2.5 times faster than the university in the above example. The *x*-index is more realistic by predicting that the probability of obtaining a Nobel Prize achievement would be 180 times higher for MIT than for the university in the example.

I did not try to generate and validate an *x*-index for economic sciences. The bases for the generation of this index are the same as in the natural sciences; the problem lies exclusively in how to record the citations. It must be noted that for highly cited papers in natural sciences, the numbers of citations in Google Scholar are about the same or even less than in the ISI Web of Science, which indicates that the ISI Web of Science has an almost universal coverage of citation in natural sciences. The same cannot be concluded for economic sciences, where the number of citations of some highly cited papers may be three or four times higher in Google Scholar than in the ISI Web of Science.

In summary, the evaluation of the level of research performance of countries and institutions by exclusively using the high-citation tail of the citation distribution is much more accurate and reliable than other types of evaluations that consider all scientific publications. The *x*-index combines simplicity of calculation and high accuracy, which is demonstrated by its high correlation with the number of Nobel Prize achievements across countries and institutions.
